# The renal angina index accurately predicts low risk of developing severe acute kidney injury among children admitted to a low-resource pediatric intensive care unit

**DOI:** 10.1080/0886022X.2023.2252095

**Published:** 2023-09-19

**Authors:** Christina Zulu, Chisambo Mwaba, Somwe wa Somwe

**Affiliations:** a Department of Paediatrics and Child Health, School of Medicine, University of Zambia, Lusaka, Zambia; b Department of Paediatrics, University Teaching Hospitals - Children’s Hospital, Lusaka, Zambia; c Beit-Cure Hospital, Lusaka, Zambia

**Keywords:** Acute kidney injury, pediatric intensive care unit, renal angina index, modified renal angina index, severe acute kidney injury, KDIGO

## Abstract

**Background:**

Acute kidney injury (AKI) increases the risk of adverse outcomes. The renal angina index (RAI) has previously been used to predict patients at risk of developing severe AKI (sAKI).

**Method:**

This single-centre prospective observational study aimed to assess the prevalence of sAKI in PICU as the primary outcome and the duration of mechanical ventilation and PICU stay, RRT need, and mortality as secondary outcomes. The utility of the RAI in predicting day 3 sAKI was also assessed. We enrolled 122 patients aged 1 month to 16 years whose baseline characteristics were collected *via* questionnaire. RAI was calculated on day 0 with a score of ≥8 being considered positive. sAKI was defined as KDIGO stages 2 and 3.

**Results:**

sAKI prevalence was 14.8% and its development was associated with longer duration of mechanical ventilation (*p* = 0.001) and higher mortality (*p* = 0.011). A positive Day 0 RAI predicted day 3 sAKI with sensitivity 55.6%, specificity 85.6%, PPV 40.0%, NPV 91.8%, and AUC of 0.77. Exclusion of children older than 5 years improved RAI performance (sensitivity 72.7%, specificity 88.0%, PPV 57.1%, NPV 93.6%, AUC 0.80). A modified RAI based on local AKI risk factors had equivalent performance to RAI (Z – score 0.78 (CI −0.077–0.033), *p* = 0.435) with sensitivity 72.2%, specificity 80.8%, PPV 39.4%, NPV 94.4% and AUC 0.80.

**Conclusion:**

The RAI can be an effective tool in ruling out sAKI in patients and a modification of RAI based on population-based risk factors improves the test’s sensitivity and NPV.

## Introduction

Acute kidney injury (AKI) is a clinical syndrome in which sudden deterioration in renal function results in the inability of the kidneys to maintain fluid and electrolyte homeostasis [1]. According to the KDIGO Acute Kidney Injury Work Group (2012), AKI can be defined as any one of the following: An increase in SCr by ≥ 26.5 μmol/l (≥0.3 mg/dl) within 48 h, or an increase in SCr to ≥1.5 times the baseline, which is known or presumed to have occurred within the prior 7 days, or a urine volume <0.5 mL/kg/h for 6 h [2].

Advances in intensive care have reduced the mortality of critically ill patients, but a concomitant increase in the incidence of AKI in the pediatric intensive care unit (PICU) has been noted [3]. AKI ranges from sub-clinical injury to severe renal failure requiring renal replacement therapy (RRT) [4]. Several studies conducted in India on the epidemiology of AKI in this group of children give a prevalence ranging from 10 to 82% [5–7]. There is limited data on the epidemiology of AKI in sub-Saharan Africa, but the prevalence is estimated to be higher than that in high-income countries (HIC) [8]. The unclear picture of AKI epidemiology can be attributed to limited resources and infrastructure for epidemiological mapping. Additionally, unavailability of nephrologists and diagnostic capacity also hamper AKI recognition [9,10].

It has been shown that even minor degrees of renal dysfunction are associated with poor outcomes [11]. Serum creatinine (SCr) is currently considered the gold standard for the diagnosis of AKI. It is an insensitive marker for early detection of AKI since it does not significantly increase until about half of the kidney function has been lost [4,7]. The concept of renal angina was proposed by Goldstein & Chawla [12] as an adjunct to assist in the early detection of AKI. They postulated that just as troponin I is used to prompt evaluation and therapeutic intervention for myocardial infarction, the renal angina index (RAI) when combined with AKI biomarkers could predict the development of AKI. The RAI combines risk factors of AKI and early signs of loss of function (rise in serum creatinine or extent of fluid accumulation) to identify patients at risk for subsequent severe AKI (sAKI). sAKI is defined as KDIGO stages 2 and 3 [13].

A multicenter study carried out by Basu et al. [14] showed that the RAI was superior to KGIDO (Kidney Disease: Improving Global Outcomes) in predicting Day 3 sAKI in critically ill children. A larger follow-up study carried out in 32 PICUs revealed similar findings with renal angina positivity being associated with a higher risk of sAKI and worse outcomes such as increased use of RRT, longer duration of mechanical ventilation, and increased mortality [13]. Studies from India and Egypt also found that the RAI predicted sAKI with sensitivities of 82.8% and 84.6%, respectively [15,16]. The utility of the RAI in predicting sAKI has never been validated in children admitted to PICUs in sub-Saharan Africa.

The main objective of this study was to determine the ability of the RAI to predict the development of day three sAKI in children admitted to the PICU. Other objectives were to determine the prevalence and risk factors of sAKI in children admitted to the PICU.

## Materials and methods

### Study design, site, and population

This was a prospective observational study carried out in the PICU at University Teaching Hospitals – Children’s Hospital (UTH-CH) in Lusaka Zambia over a period of fifteen months starting in October 2020 and ending in January 2022. UTH-CH is the largest pediatric referral hospital in Zambia with a bed capacity of 365 and has an eight (8) bed level II PICU. A recent study evaluated the use of a modified pediatric risk of mortality (PRISM) III scoring system, which is a clinical predictive model computed based on 17 commonly used physiological variables including cardiovascular, neurologic, respiratory, chemistry and heamatologic parameters and whose total score ranges from 0 to 74 with higher scores predicting greater risk of mortality [17]. The study showed that patients admitted to our PICU had a median PRISM III score of 5 [17].

The study population consisted of all children aged one month to 16 years admitted to the PICU. Neonates, children with AKI stage 2 and above, chronic kidney disease (CKD), and those with a PICU length of stay less than 72 h were excluded.

### Sample size calculation

The sample size was determined using the sample size calculation for surveys at the 0.05 level of significance. Incidence was used as a proxy for prevalence. The incidence of AKI in the PICU was 10% based on a worldwide study by Schneider et al. and the Indian study by Sundararaju et al. [15,18]. In 2019, 486 patients were admitted to the UTH – CH PICU, and correcting for this population, the final sample size was 118.

### Collection of clinical data, study variables, and laboratory procedures

Baseline participant clinical data was collected using a pre-designed questionnaire. The study’s independent variables included patient age, sex, diagnosis on admission, presence of shock, baseline laboratory parameters, and whether the patient required the use of mechanical ventilation or vasopressor support within 8 h of admission. Others were a history of bone marrow transplantation, percentage fluid overload, and baseline SCr (BSCr).

The study’s primary dependent variable was the development of sAKI. Secondary outcomes included the length of stay in the PICU, patient requirement of RRT, the duration of mechanical ventilation, and mortality.

During the first eight h of admission, four milliliters of peripheral blood was collected and taken to the laboratory for routine baseline investigations and SCr. On day 3 of PICU admission (between 72 and 96 h), two (2) milliliters of peripheral blood was sent for SCr estimation. The Jaffe method was used for creatinine analysis [7].

### Study definitions

The baseline serum creatinine (BSCr) was defined as the lowest creatinine recorded in the 90 days prior to PICU admission. If not available in the patient’s records, an eCrCl of 120 mL/min per 1.73 m^2^ was used to calculate BSCr as validated by previous studies [13,19,20].

The percentage of fluid overload (FO) was determined by assessing input and output in the first 8 h of PICU admission [15]. This time frame was beyond the accepted window of early goal-directed therapy (EGDT) of resuscitation and allowed time for some diuresis to take place [21].

%FO=[(Fluid in−Fluid out) ÷Adm weight] ×100

Change in SCr from baseline was calculated by dividing serum creatinine at enrollment (after a minimum of 8-12 h of PICU admission) by baseline serum creatinine [15].

$$\Delta \mathrm{SCr}\;=\frac{\mathrm{Enrollement}\;\mathrm{SCr}}{\mathrm{Baseline}\;\mathrm{SCr}}$$

**Figure 1.**
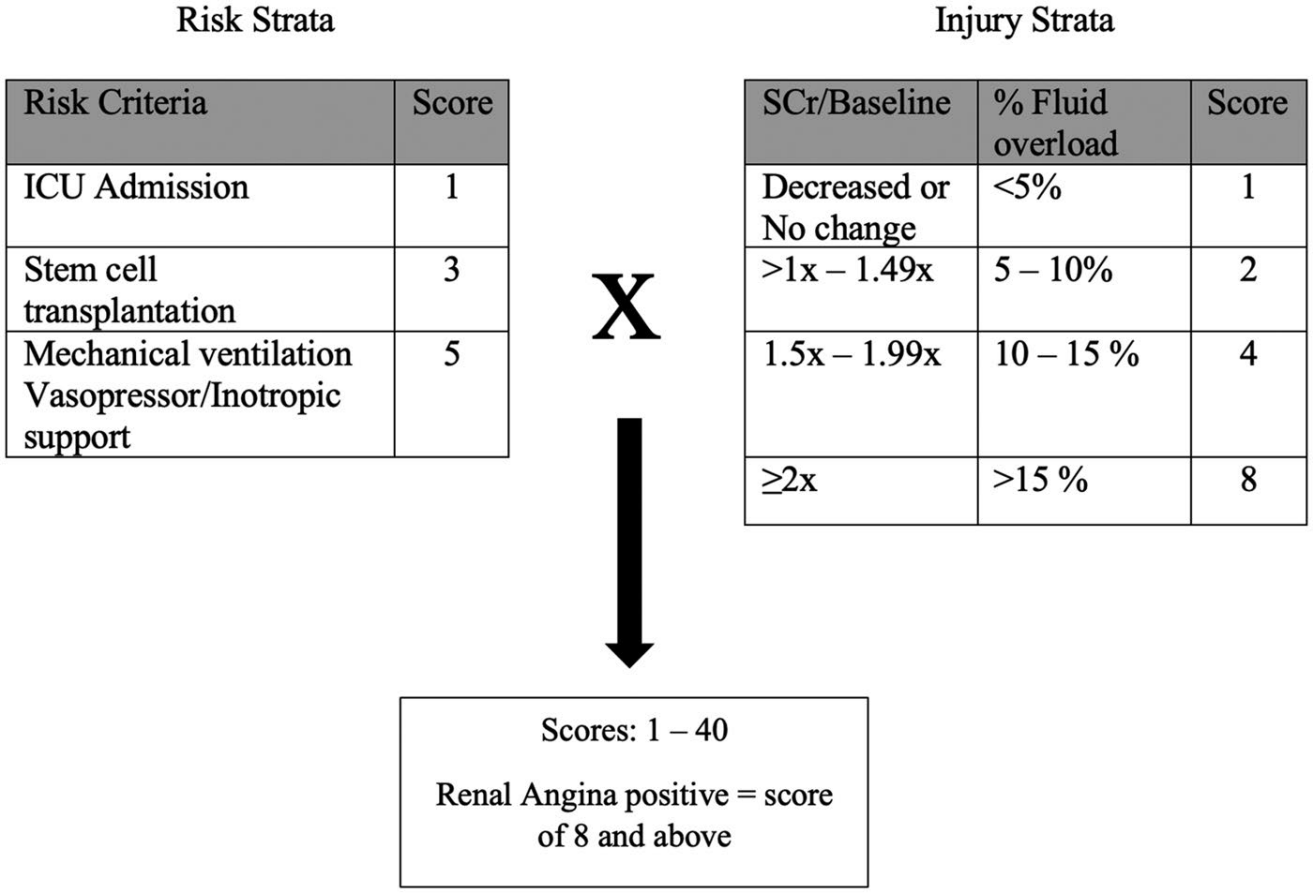
The Renal angina index.

The RAI was calculated by multiplying the Risk score by the Injury score. Patients with a RAI score ≥8 were classified as renal angina positive (RA+), and those with a score <8 were classified as renal angina negative (RA-) as shown in Figure 1.

The definition of AKI was based on the KDIGO 2012 criteria. The pediatric definition of AKI is similar to that used in adults [2].

Severe AKI was defined as KDIGO stage ≥2: Increase SCr of 200% from baseline, a decrease in eCrCl of ≥50% from baseline, or ≤0.5 mL/kg/h of urine output for ≥8 h.

### Statistical analysis

Data was analyzed using Statistical Package for the Social Sciences (SPSS) version 29 (IBM corporation, Armonk, NY). Categorical variables were expressed as simple proportions while continuous variables were expressed as medians with interquartile range if asymmetrically distributed, and as means with standard deviations if symmetrically distributed. Normality was determined using the Shapiro-Wilk test. Categorical variables were compared using Pearson’s chi-square test otherwise, Fisher’s exact test was used in circumstances where a cell contained fewer than five. The independent student’s t-test was used to compare means as a way of assessing the association between continuous variables and sAKI. For continuous variables with an asymmetric distribution, the Kruskal-Wallis test was used for comparison.

Independent variables with a p-value less than 0.1 and variables shown to have a significant correlation to sAKI were used to construct a multivariable logistical regression model to determine the predictors of the development of sAKI. The diagnostic utility of RAI was assessed using sensitivity, specificity, negative and positive predictive values (NPV and PPV), and estimates of the area under the curve (AUC).

The RAI score was modified using methods described in two studies by Matsuura et al. [22,23]. The cutoff point for the derived modified RAI (mRAI) was determined by using Youden’s index while the generated mRAI AUC was compared to the RAI AUC using non-parametric methods (z-test). Values of *p* ≤ 0.05 were considered statistically significant for all calculations.

### Ethical considerations

Ethical clearance was obtained from the University of Zambia Biomedical Research Ethics Committee (UNZABREC REF No 943-2020). Patient confidentiality was protected by anonymising the data and written informed consent was obtained from the parents/guardians of all participants before enrollment into the study.

## Results

### Baseline demographic and clinical characteristics of patients

**Figure 2.**
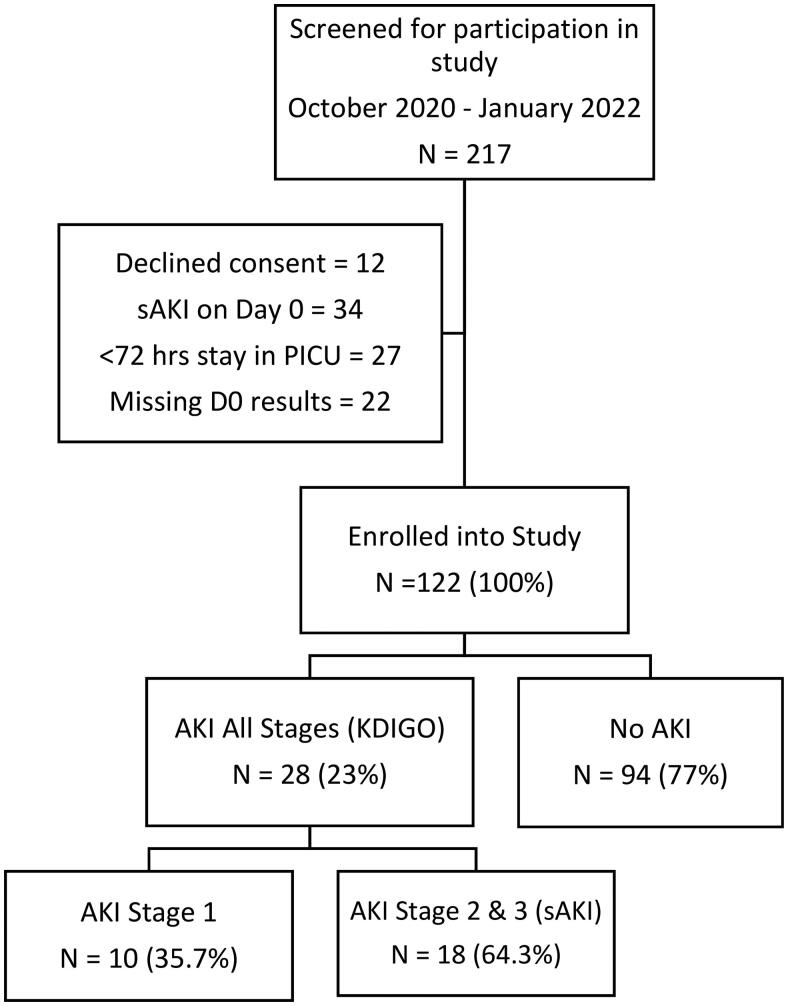
Study recruitment flow chart for sAKI.

Of the 217 children who were screened, only 122 were enrolled in the study. Reasons for exclusion from the study included refusal to consent in 12 patients, presence of sAKI on admission in 34, being admitted to PICU for less than 72 h for 27 of the patients while 22 of the children had missing results. Therefore, only 122 patients were enrolled in the study and included in the final analysis. This information is shown in Figure 2.

The median age of patients was 5.5 years (IQR 1.5–11.2). A majority of the patients were infants representing 29.5%. There were 67 (54.9%) males and 55 (45.1%) females enrolled.

The main indication leading to PICU admission was central nervous system (CNS) diagnoses in 57 (46.7%) patients, among which included viral meningoencephalitis in 20 (35.1%), status epilepticus in 9 (15.8%) and brain abscess in 8 (14.0%). This was followed by sepsis in 17 (13.9%), diabetic ketoacidosis in 10 (8.2%), cardiovascular disease in 10 (8.2%), and severe malaria in 8 (6.6%), as shown in Table 1.

**Table 1.** Clinical diagnosis of children admitted to PICU.

Primary System Disorder	N (%)
Central Nervous System	57 (46.7)
Viral meningoencephalitis	20 (35.1)
Status epilepticus	9 (15.8)
Brain abscess	8 (14.0)
Tuberculous meningitis	6 (10.5)
Bacterial meningitis	2 (3.5)
Cerebrovascular accident	2 (3.5)
Brain tumor	2 (3.5)
Intracranial hemorrhage	2 (3.5)
Guillain - Barre syndrome	1 (1.8)
Acute demyelinating encephalomyelitis	1 (1.8)
Tetanus	1 (1.8)
Hydrocephalus	1 (1.8)
Hypertensive emergency	1 (1.8)
Cavernous sinus thrombosis	1 (1.8)
Sepsis	17 (13.9)
Endocrine	12 (9.8)
Diabetic ketoacidosis	10 (83.3)
Hypoglycemia	1 (8.3)
Adrenal insufficiency	1 (8.3)
Cardiovascular	10 (8.2)
Congestive cardiac failure	6 (60.0)
Cardiac tamponade	2 (20.0)
Cardiogenic shock	1 (10.0)
Supraventricular tachycardia	1 (10.0)
Severe Malaria	8 (6.6)
Respiratory	7 (5.7)
Severe pneumonia	4 (57.1)
Foreign body inhalation	1 (14.3)
Empyema thoracis	1 (14.3)
Upper airway obstruction	1 (14.3)
Poisoning	4 (3.3)
Haematological	3 (2.5)
Hemophilia	2 (66.7)
Severe anemia in Sickle cell anemia	1 (33.3)
Gastrointestinal – Intestinal obstruction	1 (0.8)
Renal – Acute glomerulonephritis	1 (0.8)
Surgical – Post operative (laparotomy)	1 (0.8)
Multisystem inflammatory syndrome in children	1 (0.8)

Twenty-three (18.9%) of the enrolled patients required mechanical ventilation, and of these, only 21 (91.3%) received ventilation (Table 2). The rest of the children (2, or 8.7%) were not ventilated due to the non-availability of a free mechanical ventilator at the time of admission. The mean duration of mechanical ventilation was 2.44 days (±9.8) while the majority of patients, 102 (83.6%) were ventilated for 3 days or less.

**Table 2.** Baseline participant characteristics compared by sAKI status.

Parameter		Overall	sAKI No (%)	No sAKI No (%)	P value
		122	18 (14.8)	104 (85.2)	
Sex	Male	67 (54.9)	7 (38.9)	60 (57.7)	0.139
	Female	55 (45.1)	11 (61.1)	44 (42.3)	
Age	Median	5.5 years (IQR 1.6–11.3)	1.4 years (IQR 0.3–10.8)	6.0 years (IQR 2–11.8)	0.064
Age					0.006
	< 1 year	36 (29.5)	11 (61.1)	25 (24.0)	
	2 – 5 years	25 (20.5)	3 (16.7)	25 (24.0)	
	6 – 10 years	28 (23.0)	0 (0)	25 (24.0)	
	≥ 11 years	33 (27.0)	4 (22.2)	29 (27.9)	
Primary System dysfunction/ Diagnosis					0.010
	CNS	57 (46.7)	4 (22.2)	53 (51.0)	
	Sepsis	17 (13.9)	7 (38.9)	10 (9.6)	
	Endocrine	12 (9.8)	1 (5.6)	11 (10.6)	
	Cardiovascular	10 (8.2)	0	10 (9.6)	
	Severe Malaria	8 (6.6)	2 (11.1)	6 (5.8)	
	Respiratory	7 (5.7)	2 (11.1)	5 (4.8)	
	Other	11 (9.0)	2 (11.1)	9 (8.6)	
Δ SCr					0.0001
	No change	39 (32)	2 (11.1)	37 (35.6)	
	>1x–1.49x	43 (35.2)	3 (16.7)	40 (38.5)	
	1.5x–1.99	36 (29.5)	9 (50.0)	27 (26.0)	
	≥2x	4 (3.3)	4 (22.2)	0	
Elevated D0SCr					0.054
	Yes	82 (67.2)	16 (88.9)	66 (63.5)	
	No	40 (32.8)	2 (11.1)	38 (36.5)	
Transplant					*
	Yes	0	0	0	
	No	122 (100)	18 (100)	104 (100)	
Mechanical Ventilation					0.0497
	Yes	21 (17.2)	6 (33.3)	15 (14.4)	
	No	101 (82.8)	12 (66.7)	89 (85.6)	
Vasopressor support					0.089
	Yes	23 (18.9)	6 (33.3)	17 (16.3)	
	No	99 (81.1)	12 (66.7)	87 (83.7)	
RAI Positive					<0.001
	Yes	25 (20.5)	10 (55.6)	15 (14.4)	
	No	97 (79.5)	8 (44.4)	89 (85.6)	
Parameter		Overall	sAKI	No sAKI	P value
Baseline Serum Creatinine	Median (µmol/L)	35.1 (IQR 26.3–44.9)	28.7 (IQR 19.9–45.0)	35.4 (IQR 26.6–45.0)	0.083
Day 0 Serum Creatinine	Median (µmol/L)	37.5 (IQR 28.8–57.1)	48.0 (IQR 32.8–64.0)	35.2 (IQR 26.7–57.0)	0.128
Day 3 Serum Creatinine	Median (µmol/L)	32.85 (IQR 22.7–50.6)	81.9 (IQR 54.0–153.6)	28.9 (IQR 21.5–41.5)	<0.001
Length of PICU stay					0.156
	≤3 days	30 (24.6)	5 (27.8)	25 (24.0)	
	4 – 7 days	51 (41.8)	4 (22.2)	47 (45.2)	
	≥8 days	41 (33.6)	9 (50)	32 (30.8)	
Median length of stay					
	Days	6 (IQR 4 – 9)	8 (IQR 3 – 10)	6 (IQR 4 - 9)	0.479
Number of days on Ventilation	*N* = 28				0.384
	≤3 days	8 (28.6)	3 (30.0)	5 (27.8)	
	4 – 7 days	10 (35.7)	5 (50.0)	5 (27.8)	
Median duration of ventilation	≥8 days	10 (35.7)	2 (20.0)	8 (44.4)	
	Days	0 (IQR 0 - 0)	3 (IQR 0–5.3)	0 (IQR 0 - 0)	0.001
RRT					0.148
	Yes	1 (0.8)	1 (5.6)	0	
	No	121 (99.2)	17 (94.4)	104 (100)	
Mortality					0.011
	Yes	20 (16.4)	7 (38.9)	13 (12.5)	
	No	102 (83.6)	11 (61.1)	91 (87.5)	

Twenty-three (18.9%) patients received vasopressor support. The extent of fluid overload could not be reliably estimated because 75 (61.5%) of the recruited patients did not have documented fluid input and output during the first 8 h of admission to the PICU. On admission, the RAI was positive in 25 (20.5%) of the patients (Table 2). None of the study participants had a prior history of hematopoietic stem cell transplant.

### Prevalence of severe AKI and patient outcomes

Renal function tests showed a median BSCr of 35.1 µmol/L (IQR 26.3–44.9) and a median day 0 SCr (D0SCr) of 37.5 µmol/L (IQR 28.8–57.1). Thirty-nine (32%) patients had no change in SCr from baseline, 43 (35.2%) had *a* > 1–1.49 times increase, and 36 (29.5%) had a 1.5–1.99 times increase from baseline. Only 4 (3.3%) patients had *a* ≥ 2 times increase in SCr from baseline.

The Day 3 SCr (D3SCr) was at a lower median value of 32.85 µmol/L (IQR 22.70–50.55) compared to the median D0SCr. Based on the KDIGO 2012 criteria, only 28 (23%) patients developed AKI. Of these, 18 were classified as sAKI, giving a prevalence of 14.8%. Only one (0.8%) patient received RRT, while the rest of the patients with AKI were managed conservatively.

The average length of stay in PICU was 6 days (IQR, 3.75–9.25 days). The PICU length of stay was ≤ 3 days for 30 (24.6%) of the patients, between 4–7 days for 51 (41.8%) of the patients, and ≥ 8 days for 41 (33.6%) of the patients. Of the patients that spent more than 8 days in the PICU, 4 of them were admitted for longer than 22 days.

A total of 20 (16.4%) patients died while still admitted to the PICU. The remaining 102 (83.6%) patients were successfully discharged and transferred to the general wards. This information has been depicted in Table 2.

### Association of baseline characteristics with severe AKI

Table 2 shows that eighteen (14.8%) patients developed sAKI. Of these, 7 (38.9%) were male, while 11 (61.1%) were female (*p* = 0.139). The median age of the patients [with sAKI 1.4 years (IQR 0.28, 10.75) vs without sAKI 6 years (IQR 2, 11.75), *p* = 0.064] was not significantly different between the two groups.

The leading cause of admission was sepsis (*n* = 7, 38.9%), followed by CNS dysfunction (*n* = 4, 22.2%) in the sAKI group. The median BSCr and D0SCr were comparable in the two groups with a notable difference in D3 SCr [81.9 µmol/L (IQR 54.0, 153.6) vs. 28.9 µmol/L (IQR 21.5, 41.5), p = <0.001].

Ten (55.6%) of the patients who developed sAKI were RA positive, which gave a p-value of 0.0001. Change in SCr from baseline was the only RAI parameter that showed significance, with 88.9% of sAKI patients having an elevated SCr compared to 63.5% of non-sAKI patients (*p* = 0.0001). Percentage of FO (*p* = 1.000), use of mechanical ventilation (33.3% vs 14.4%, *p* = 0.084) and administration of vasopressors (33.3% vs 16.3%, *p* = 0.106) did not appear to affect the development of sAKI.

The development of sAKI was associated with a longer duration of mechanical ventilation [3 (IQR, 0–5.3) days, *p* = 0.001] and a higher mortality rate (*n* = 7, 38.9%, *p* = 0.011) compared to the non-sAKI group. Only 1 patient with sAKI received RRT (5.6%, *p* = 0.148) and the duration of PICU stay was 7.5 (3–10.25) days in the sAKI group and 6 (4 – 9) days in the non-sAKI group (*p* = 0.479).

### Predictors of sAKI

Independent variables were selected for logistic regression based on the criteria described in the methods section. A positive RAI predicted the development of sAKI on day three, with a UOR of 25.7 (95% CI: 2.0, 331.8, *p* = 0.013) and an AOR of 6.5 (95% CI: 2.0, 21.0, *p* = 0.002) in the model, and a diagnosis of sepsis increased the odds of developing sAKI on day three by 16.9 (95% CI: 1.8, 160.1, *p* = 0.014) in the UOR and 6.6 (95% CI: 1.5, 2.9, *p* = 0.013) in the AOR. This information is depicted in Table 3.

**Table 3.** Regression analysis of clinical parameters of patients admitted to PICU.

Parameters		Odds Ratio (95% CI)	P value	Adjusted Odds Ratio (95% CI)	P value
Age		0.95 (0.8–1.1)	0.403		
Sex	Male	Ref			
	Female	2.3 (0.6–9.2)	0.232		
Diagnosis	CNS	Ref		Ref	
	Sepsis	16.9 (1.8–160.1)	0.014	6.6 (1.5–29.8)	0.013
	Respiratory	1.3 (0.1–14.1)	0.843	1.1 (0.2–5.6)	0.904
	Severe Malaria	7.8 (0.6–96.0)	0.107	4.4 (0.6–35.7)	0.157
	Other				
		4.7 (0.5–49.8)	0.196	5.4 (0.7–39.8)	0.101
D0SCr	µmol/L	1.0 (1.0–1.1)	0.806		
ΔSCr	No	1			
	Yes	1.3 (0.2–10.6)	0.827		
Vasopressor support	No	Ref			
	Yes	0.1 (0.0–1.2)	0.069		
Ventilation	No	Ref			
	Yes	0.5 (0.1–5.5)	0.606		
RAI Positive	No	Ref		Ref	
	Yes	25.7 (2.0–331.8)	0.013	6.5 (2.0–21.0)	0.002

### Diagnostic precision of the renal angina index

A positive RAI on day 0 was found to have a sensitivity of 55.6%, a specificity of 85.6%, a PPV of 40.0%, and an NPV of 91.8%, as shown in Table 4. When RAI was considered positive at a score of 10 and above, the sensitivity decreased to 44.8% and the NPV to 89.9%, whereas the specificity remained the same. At a cutoff of 12, the sensitivity decreased further to 33.6%, whereas the specificity and PPV increased to 94.2% and 50%, respectively.

**Table 4.** RAI Sensitivity, specificity, NPV, and PPV.

Criteria	Sensitivity % (95% CI)	Specificity % (95% CI)	PPV % (95% CI)	NPV % (95% CI)
D0SCr > BSCr	88.9(74.4–103.4)	36.5(27.3–45.8)	19.5(10.9–28.1)	95.0(88.2–101.8)
RAI score ≥ 8	55.6(32.6–78.5)	85.6(78.8–92.3)	40.0(20.8–59.2)	91.8(86.3–97.2)
RAI Score ≥ 10	44.4(21.5–67.4)	85.6(78.8–92.3)	34.8(15.3–54.2)	89.9(84.0–95.8)
RAI score ≥ 12	33.6(11.6–55.1)	94.2(89.8–98.7)	50.0(21.7–78.3)	89.1(83.3–94.9)

**Figure 3.**
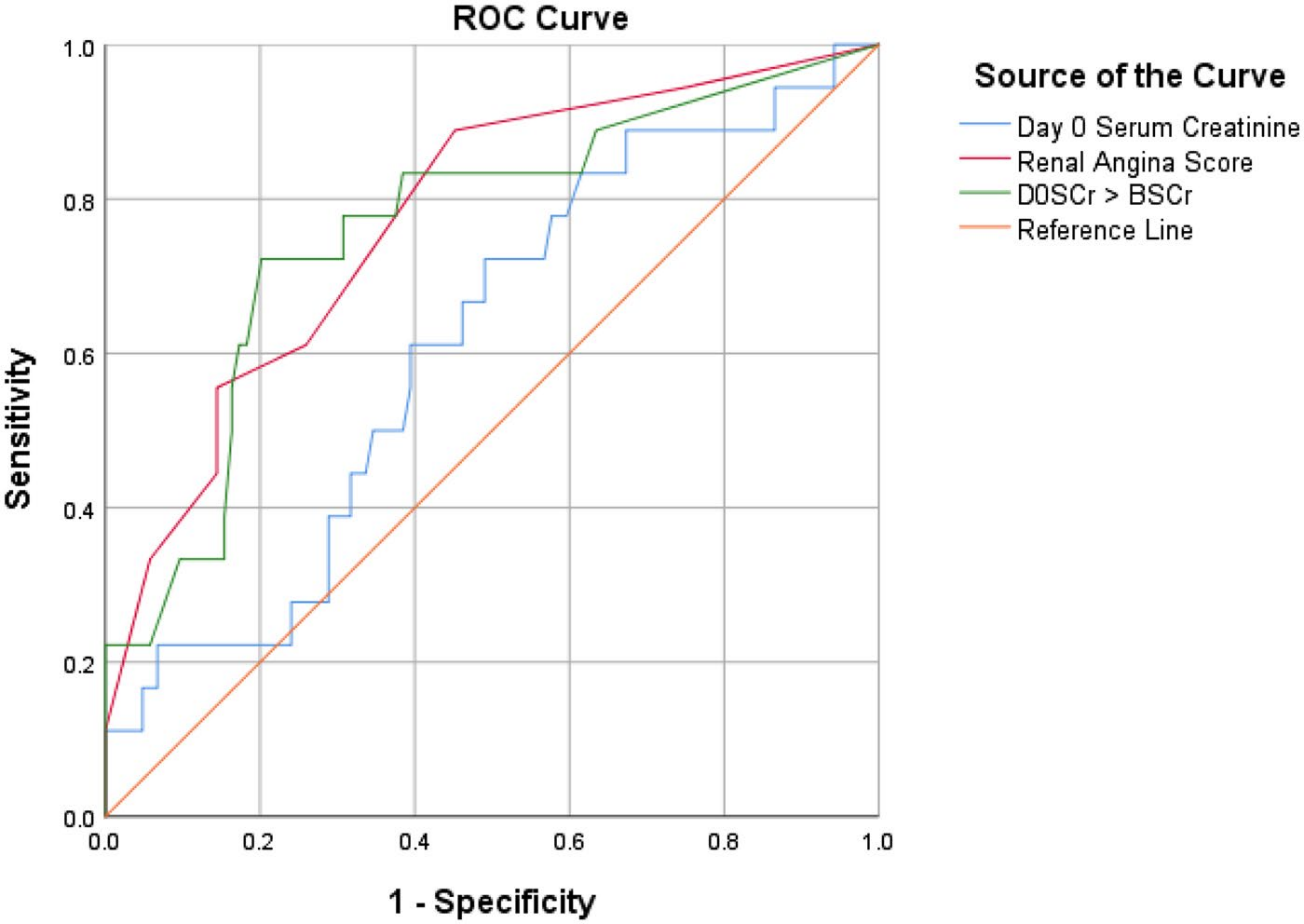
Receiver Operating Characteristic (ROC) curves for RAI, D0SCr, and ΔSCr for predicting severe AKI on Day 3.

The receiver operating characteristic (ROC) curve to determine how predictive the RAI score was at D0 for sAKI indicated an area under the curve (AUC) of 0.77 (77%), 95% CI: 0.66, 0.89 at *p* = 0.0001 (X^2^ test value of 18.8), while D0SCr was 0.61 (61%), CI: 0.48, 0.75, and ΔSCr was 0.76 (76%), 95% CI: 0.64, 0.89. Based on the obtained AUC, the RAI was considered a ‘fair’ test. The above information is shown in Table 5 and Figure 3.

**Table 5.** Diagnostic precision of Renal angina index.

Test Variable	AUC	95% Confidence Interval	Significance level
RAI	0.77	0.66–0.89	0.0001
D0 SCr	0.61	0.48–0.75	0.130
ΔSCr	0.76	0.64–0.89	0.001

This indicated that the RAI is superior to D0SCr in predicting the development of sAKI on day 3 with a statistically significant difference of 0.162, 95%, CI: 0.05, 0.28 at *p* = 0.006. The RAI also performed better than ΔSCr; however, the sensitivity difference was statistically insignificant (0.012, 95% CI: −0.9.0.12, *p* = 0.82).

**Figure 4.**
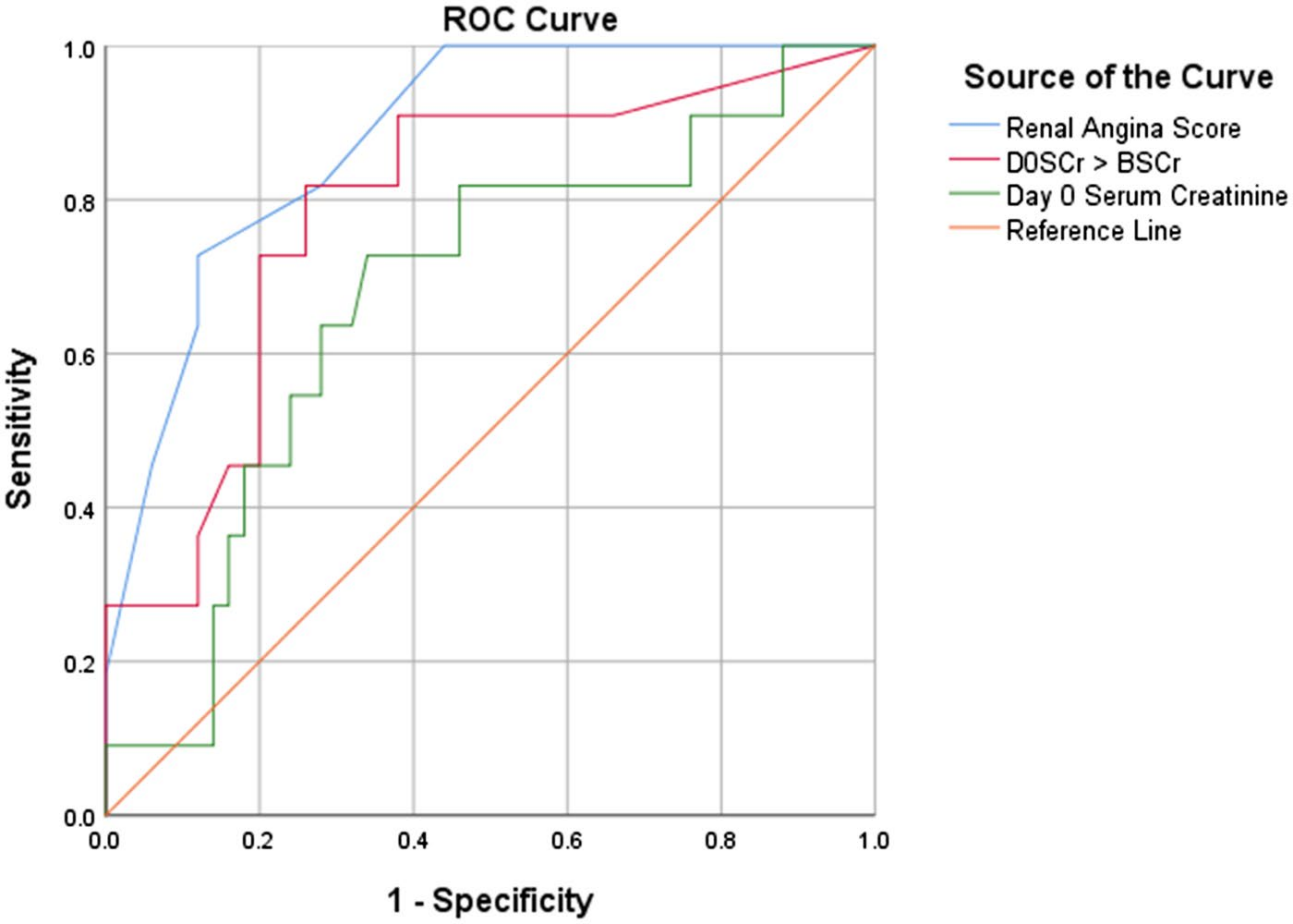
Receiver Operating Characteristic (ROC) curves for RAI, D0SCr, and ΔSCr for predicting severe AKI on Day 3 in children ≤5 years.

Since 72% (13/18) of sAKI patients were < 5 years old a sub-analysis of the predictive value of RAI in this age group was undertaken. Among children ≤ 5 years of age the RAI sensitivity, specificity, PPV, NPV, and AUC were 72.7%, 88.0%, 57.1%, 93.6%, and 0.88 respectively. This information is depicted in Tables 6, 7 and Figure 4.

**Table 6.** RAI Sensitivity, specificity, NPV, and PPV in children ≤5 years.

Criteria	Sensitivity % (95% CI)	Specificity % (95% CI)	PPV % (95% CI)	NPV % (95% CI)
D0SCr > BSCr	90.9(73.9–107.9)	34.0(20.9–47.1)	23.3(10.6–35.9)	94.4(83.9–105.0)
RAI score ≥ 8	72.7(46.4–99.0)	88.0(79.0–97.0)	57.1(31.2–83.1)	93.6(86.7 - 100)

**Table 7.** Diagnostic precision of Renal angina index in children ≤5 years.

Test Variable	AUC	95% Confidence Interval	Significance level
RAI	0.88	0.79–0.98	0.0001
D0 SCr	0.66	0.50–0.85	0.07
ΔSCr	0.79	0.64–0.94	0.003

### Modified renal angina index (mRAI) based on local AKI risk factors

**Figure 5.**
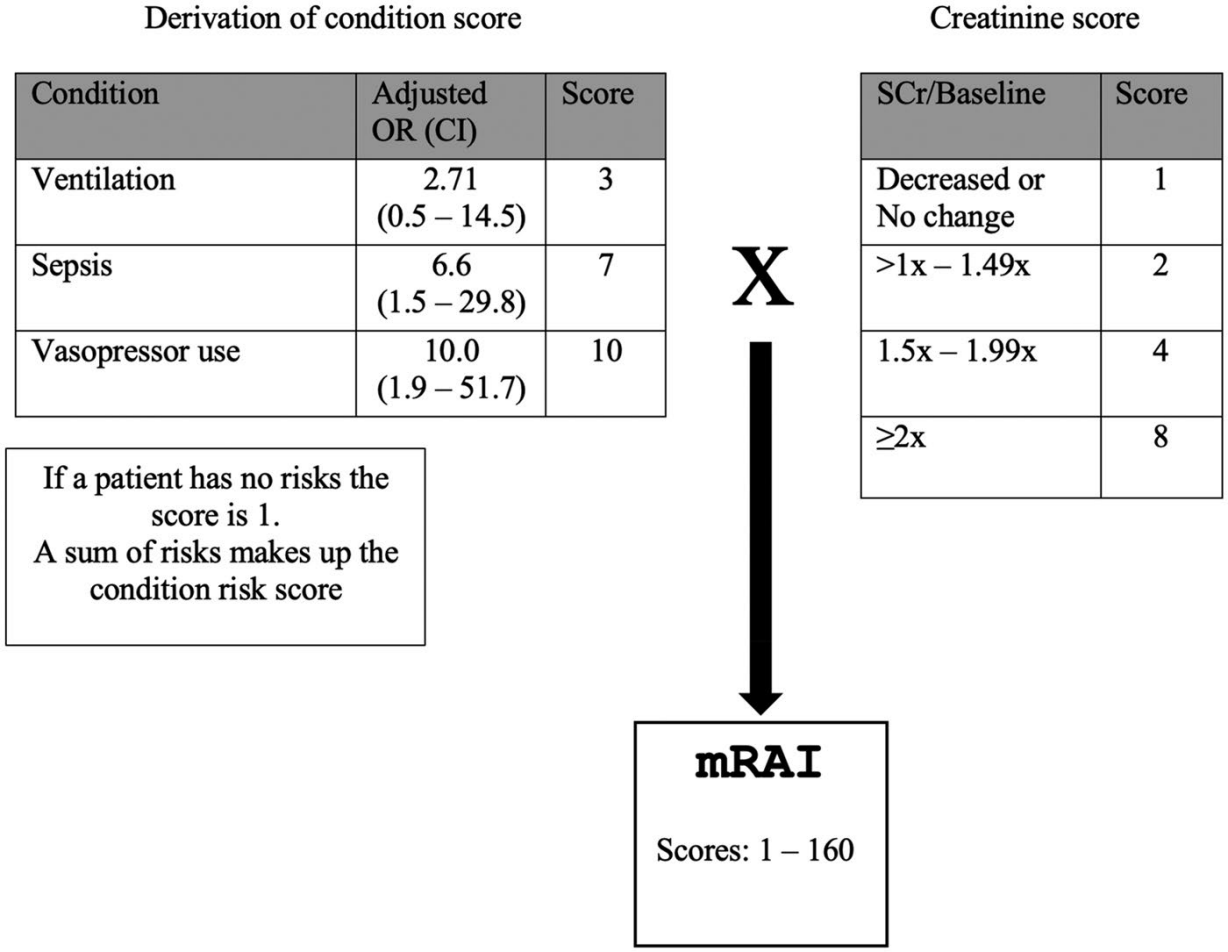
Modified RAI based on local risk factors.

We attempted to modify the RAI by using risk factors established in an earlier study conducted in our PICU as well as in the present cohort using methods described by Matsuura et al. [22–24]. The mRAI was calculated by multiplying the sum of the risk scores by the original RAI creatinine score and this gave scores of 1, 2, 3, 4, 6, 7, 8, 10, 12, 13, 14, 17, 20, 24, 26, 28, 34, 40, 56, 68, 80, 104, 136, and, 160 as shown in Figure 5.

**Figure 6.**
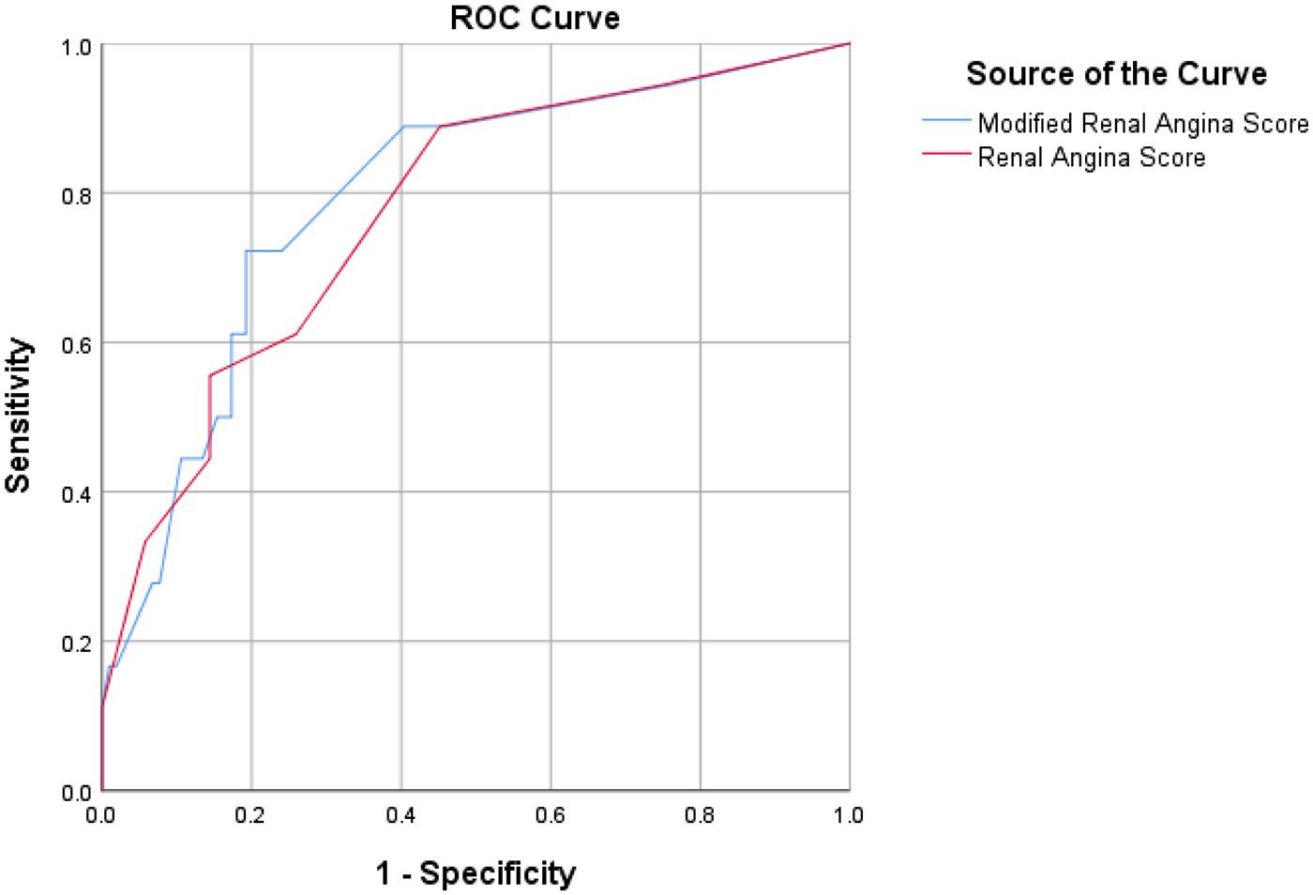
Receiver Operating Characteristic (ROC) curves for modified RAI and original RAI.

This score had an improved performance of AUC 0.80 to predict sAKI on day 3 (Figure 6 and Table 8). This AUC, however, was not statistically different when compared to the AUC generated using the original RAI (z = −0.02 (CI −0.07–0.03), *p* = 0.435) as is seen in Table 9.

**Table 8.** Diagnostic precision of modified Renal angina index.

Test Variable	AUC	95% Confidence Interval	Significance level
RAI	0.77	0.66–0.89	0.0001
mRAI	0.80	0.68–0.91	0.0001
D0 SCr	0.61	0.48–0.75	0.130
ΔSCr	0.76	0.64–0.89	0.001

**Table 9.** Paired-Sample Area difference under the ROC curves for mRAI and RAI.

Test Result Pair(s)	Asymptotic		AUC Difference	Std. Error Difference^b^	Asymptotic 95% Confidence Interval	
	Z	Sig. (2-tail)^a^			Lower Bound	Upper Bound
RAI_score - mRAI_score	−0.781	0.435	−0.022	0.334	−0.077	0.033

Upon validation within this cohort, the mRAI was tested at various cutoff points with Youden’s index calculated. This gave an optimum cutoff point of ≥ 8 (Table 10).

**Table 10.** Modified RAI sensitivity, specificity, NPV, PPV, and youden’s index at various cutoff points.

Cut off Point	Sensitivity % (95% CI)	Specificity (95% CI)	PPV (95% CI)	NPV (95% CI)	Youden’s index
≥ 4	88.9(74.4–103.4)	59.6(50.2–69.9)	27.6(16.1–39.1)	96.9(92.6–101.1)	0.49
≥ 6	72.2(51.5–92.9)	76.0(67.7–82.2)	34.2(19.1–49.3)	94.0(89.0–99.1)	0.42
≥ 8	**72.2** (51.5–92.9)	**80.8** (73.2–88.3)	**39.4** (22.7–56.1)	**94.4** (89.6–99.2)	**0.53**
≥ 10	61.1(38.6–83.6)	80.8(73.2–88.3)	35.5(18.6–52.2)	92.3(86.8–97.8)	0.42
≥ 12	61.1(38.6–83.6)	82.7(75.4–90.0)	37.9(20.3–55.6)	92.5(87.1–97.8)	0.44
≥ 14	50.0(26.9–73.1)	83.7(76.5–90.8)	34.6(16.3–52.9)	90.6(84.8–96.6)	0.34
≥ 20	44.4(21.5–67.4)	86.5(80.0 − 93.1)	36.4(16.3–56.5)	90.0(84.1–95.9)	0.31
≥ 28	38.9(16.4–61.4)	90.4(84.7–96.1)	41.2(17.8–64.6)	89.5(83.7–95.4)	0.29
≥ 40	27.8(7.1–48.5)	93.3(88.5–98.1)	41.7(13.8–69.6)	88.2(88.2–94.2)	0.21

## Discussion

This study was conducted to determine the prevalence of sAKI, the risk factors of sAKI, and the diagnostic precision of the RAI in predicting sAKI in a low-resource PICU. Clinical characteristics of the enrolled patients were recorded on day 0 of admission to the PICU and were used to calculate RA fulfillment. Like most of the studies from low-resource countries, there were no patients with a history of stem cell transplant as this treatment option is currently not available in Zambia [25].

The proportion of children requiring mechanical ventilation and vasopressors was generally lower than has been reported from other populations. This disparity may be due to differences in the criteria utilized to select study participants, differences in morbidity patterns and may also be a reflection of limitation in PICU resources [15,26–28]

In our cohort, RA positivity was 20.5% at baseline, similar to other studies (17.9 to 22.5%) that had enrolled patients with respiratory and post-operative conditions as the indication for PICU admission [13,29]. In contrast, two American studies by Gorga et al. and Stanski et al. which only enrolled patients with septic shock reported higher RA positivity rates of 55% and 54.6%, respectively [28,30]

### Prevalence of sAKI

The sAKI prevalence (14.8%) reported in our cohort is lower than the reported sAKI burden from other centers [27,30]. Differences in the severity of illness between patients in these various cohorts could explain this discrepancy. A study by Stanski et al. that enrolled only patients with septic shock demonstrated a PRISM III score of 10.2, while, in a multicenter, multinational study from the USA, patients who developed sAKI had a median PRISM III score of 7 [13,28]. We did not calculate the baseline severity of illness scores in this cohort. However, a recent study done one year prior in our PICU, reported a median modified PRISM III score of 5 [17].

Additionally, differences in study design and patient selection could account for variations in sAKI prevalence. A study conducted in the emergency pediatric unit of a Nigerian tertiary hospital revealed a 24.1% prevalence of sAKI. In this case, the higher prevalence could be explained by the fact that patients who had already developed sAKI on admission to PICU were not excluded from the analysis as we did [31]. Similarly, a study done in China that only recruited septic shock patients showed a much higher sAKI prevalence of 54.5% [27]. Patients with sepsis tend to have higher illness severity scores and are at a higher risk of AKI [32]. Differences in the age of the enrolled study participants could also explain the disparity in sAKI prevalence as younger age has been shown to correlate to an increased rate of developing sAKI [33]

The only identified risk factors for sAKI were sepsis and RA positivity similar to findings in studies conducted among Indian, Brazilian, and Jordanian children though additional factors such as history of transplantation, younger age, male sex, use of vasopressors and mechanical ventilation, and multiorgan dysfunction were also cited in these other reports [3,26,34,35].

### Diagnostic precision of the RAI

The RAI combines risk factors with early signs of AKI on the day of admission to identify patients at increased risk of developing sAKI during their course of PICU admission. Additionally, the RAI was designed to have an extremely high NPV to effectively exclude a subgroup of patients that are not at risk of developing sAKI [38]. The RAI predicted the development of sAKI with an AUC of 0.77 (0.66–0.89). This performance was considered ‘fair’ and closely resembled findings in the second cohort of the derivation and validation study by Basu et al. in which a sensitivity of 58%, specificity of 90%, PPV of 39%, NPV of 95%, and an AUC of 0.74 were reported [21]. Our patients had similar characteristics to those of the derivation cohort.

The RAI did not perform as well as it had in other studies. Stanski et al. found that RAI predicted sAKI with a sensitivity of 98%, specificity of 54%, PPV of 31%, NPV of 99%, and AUC of 90% [37]. Similarly, a European study revealed that the RAI predicted sAKI with a sensitivity of 100%, specificity of 97%, PPV of 60%, NPV of 100%, and AUC of 0.93 [36]. The high NPV rules out AKI in patients and allows interventions such as fluid administration to proceed unhindered [14]. In our context, RAI could be calculated by the doctor admitting a patient to the PICU and used to identify patients at low risk of developing sAKI who could then continue to receive normal resuscitation and treatment. One drawback is that laboratory results for creatinine are only available after 8 h. To make this proposed intervention, especially useful point-of-care creatinine machines are advocated for so as to expedite access to the creatinine result.

Overall, RAI was better at predicting sAKI than D0SCr which predicted sAKI with an AUC of only 0.61 (0.48–0.75). Similar findings were reported by Sundararaju et al. (AUC of 0.90 vs 0.68) and Huang et al. (AUC of 0.78 vs 0.70) [15,27].

An attempt to improve the performance of the RAI was made by raising the cutoff points to ≥10 and ≥12 as shown in previous studies [15,22]. Raising the cutoff point to 10 reduced the sensitivity and NPV while specificity remained the same. Raising it to 12 further reduced the sensitivity and NPV while increasing the specificity and PPV. This finding contrasts against what was seen in the study by Sundararaju et al. where increasing the cutoff to 12 improved the performance of the RAI [15].

It was noted that in our cohort the number of AKI cases under 5 years of age was significantly higher (72%, 13/18) than described in previous studies. Therefore, a sub-analysis where all children > 5 years of age were excluded was done. It was found that the RAI performed better in children ≤5 years.

Further, a modification of RAI by adjusting the condition risk scores based on local AKI risk factors was performed. This analysis showed that the performance of the mRAI is equivalent to that of the original RAI but with improved sensitivity and NPV for our population.

One way in which the performance of RAI could be improved and especially for the assessment of children whose parents are reluctant for blood draws could be to re-emphasize the importance of monitoring urine output during the initial resuscitation period in the emergency room and also periodic weighing of children once admitted to PICU to optimize detection of fluid overload. One way this could be done is by introducing more prominent fluid flow charts to the resuscitation areas and training staff on the importance of providing this information. A possible drawback in many low-resource regions would be inadequate staff to effect this level of monitoring. One obvious advantage of weighing and urine output monitoring is that they provide readily available results as compared to serum creatinine in which there is often a lag before laboratory results become available to the attending physician.

### Outcomes of sAKI

Dialysis requirement was low with only one patient (5.6%) with sAKI receiving RRT. A previous study in the same PICU reported only 13.3% of AKI patients required RRT [24]. In contrast, dialysis requirements were higher in cohorts from the UK and China with 30.3% and 36.1% of PICU patients receiving RRT respectively [27,30]. Higher illness severity in these cohorts could explain the discrepancy. Secondly, during the period that this study was conducted, there was a shortage of dialysis consumables at our facility due to the effects of the COVID-19 pandemic.

Patients with sAKI had a significantly longer mean duration of mechanical ventilation (3 days, IQR, 0–5.3) compared to the non-sAKI group. This is a feature that was constant in the reviewed literature although the average length of ventilation reported in most studies was 6 to 9 days. This may be the result of higher illness severity among these patients compared to our cohort [3,36].

Mortality among patients with sAKI was three times higher than the mortality rate among non-sAKI patients (*p* = 0.011). Patients with sAKI tended to be more ill as evidenced by a higher rate of sepsis and subsequent use of vasopressor support and mechanical ventilation in this group of patients. These findings are similar to reports from PICU studies in the USA where mortality was found to be 37% and 20% respectively, among patients who developed sAKI [34,37]. Huang et al. carried out a similar study where they assessed the ability of the RAI to predict sAKI in 66 patients with septic shock. In contrast to our study, they found a much higher mortality of 69.4% [27]. The increased use of fluids related to medical interventions associated with septic shock that are known to increase fluid overload and mortality may explain this difference. On the other end of the spectrum, mortality was 7.7% among children with sAKI in a UK study. In this cohort, 60% of the patients in the sAKI group were elective post-cardiac surgery, and it is common practice to restrict intravenous fluids in this group. This may have reduced fluid overload, thereby decreasing the risk of mortality [29].

This study had some limitations. Firstly, not all patients admitted to PICU had fully documented emergency room urine output and fluid boluses. This led to an underestimation of the degree of fluid overload in patients and, possibly, an underestimation of RAI positivity. The poor documentation of urine output could have also led to some cases of sAKI being missed as SCr was the only parameter used to make the diagnosis of sAKI on day 3.

Secondly, this study was conducted during the second and third waves of the COVID-19 pandemic, which led to a shortage of dialysis supplies. This led to an underestimation of the need for RRT. Lastly, most patients did not have a BSCr reading, necessitating its calculation using an eCrCl of 120 mL/min/1.73m^2^ and the patient’s height.

## Conclusions

The prevalence of sAKI in children admitted to PICU is 14.8%, which is comparable to other settings. Identified risk factors for the development of sAKI included RA positivity and the presence of sepsis on admission to the PICU. sAKI is associated with a longer duration of mechanical ventilation and an increased mortality rate.

The RAI predicts sAKI in children admitted to the PICU fairly with an AUC of 0.77. It performed better when used in children ≤ 5 years old and when local AKI risk factors were used to create a modified score, the NPV and sensitivity improved but with equivalent performance. The high NPV associated with RAI/mRAI reliably ruled out AKI and can be used to screen patients in low-resource settings where it could influence decisions on change in the initial targets of resuscitation, such as fluid goals and the use of nephrotoxic medications. Additionally, RAI could be used to tag patients who would benefit from renal biomarker testing to avoid indiscriminate use of resources.

## Data Availability

The data that support the findings of this study are available from the corresponding author, C. Z., upon reasonable request.
